# Determination of Local Anesthetic Drugs in Human Plasma Using Magnetic Solid-Phase Extraction Coupled with High-Performance Liquid Chromatography

**DOI:** 10.3390/molecules27175509

**Published:** 2022-08-27

**Authors:** Shan-Yan Liang, Fang Shi, Yong-Gang Zhao, Hong-Wei Wang

**Affiliations:** 1 Hwa Mei Hospital, University of Chinese Academy of Sciences, Ningbo 315010, China; 2 Department of Chemistry, Zhejiang University, Hangzhou 310027, China; 3 College of Biological and Environmental Engineering, Zhejiang Shuren University, Hangzhou 310015, China; 4 Tongde Hospital of Zhejiang Province, Hangzhou 310012, China

**Keywords:** HPLC, MSPE, local anesthetic drugs, human plasma

## Abstract

In this work, magnetic tetraethylenepentamine (TEPA)-modified carboxyl–carbon nanotubes were synthesized, characterized, and used as adsorbents to conduct magnetic solid-phase extraction (MSPE) for the preconcentration of seven local anesthetic drugs (procaine, lidocaine, mepivacaine, oxybuprocaine, bupivacaine, tetracaine, and cinchocaine) from human plasma. The separation and determination of analytes were performed on high-performance liquid chromatography with UV detection. Several factors affected the extraction efficiency, such as the amount of adsorbents used, extraction time, sample pH, and optimization of elution conditions. Under optimal conditions, satisfactory linear relationships were obtained in the range of 0.02–5.00 mg/L, with the limits of detection (LOD) ranging from 0.003 mg/L to 0.008 mg/L. The recoveries of analytes for spiked human plasma were in the range of 82.0–108%. Moreover, the precision with intra-day and inter-day RSD values were obtained in the range of 1.5–7.7% and 1.5–8.3%. The results indicated that this method could determine the concentration of seven local anesthetic drugs in human plasma with high precision and repeatability and provide support for the clinical monitoring of the concentration of local anesthetic drugs in human plasma.

## 1. Introduction

In recent years, more and more local anesthetic drugs have been used to reversibly block nerve function [1], and some local anesthetic drugs have also been applied for postoperative analgesia and anti-arrhythmia. Structures of seven local anesthetic drugs are shown in Figure S1 from Supplementary Materials; the local anesthetic drugs can be divided into two categories (ester-type and amide-type) according to different functional groups [2]. Clinically, local anesthetic drugs are classified into short-acting, medium-acting, and long-acting local anesthetics according to the duration of action. Although local anesthetic drugs are widely used, their most common complications, including central nervous system (seizures) and cardiac (conduction disorders, cardiac arrests) toxicity, cannot be ignored. The toxicity of local anesthetic drugs and the plasma concentration appear to have some correlation, and various fatal toxication cases of local anesthetic drugs have been reported [3,4,5]. Furthermore, as emerging pollutants, their presence in effluent and environmental water is of considerable interest because of the potential for the contamination of groundwater and drinking water supplies, which may produce health risks. Therefore, the challenges that need to be resolved are the low concentrations of studied analytes and interfering substances in the complex matrix. Therefore, there is an increasing demand for a sensitive, accurate, and rapid method to determine local anesthetic drugs. Up to now, analysis methods to determine the anesthetic drugs from several sample matrices have been reported [6,7,8,9]. In order to improve the sensitivity of detection, various pretreatment methods were developed to extract local anesthetic drugs or create a cleanup matrix, such as solid-phase extraction (SPE) [10], liquid–liquid extraction (LLE) [11,12,13], and microextraction in a packed syringe (MEPS) [8,14,15].

Carbon nanotubes have been widely applied to preconcentrate, extract, and adsorb organic and inorganic analytes because of their unique features, such as large surface area and unique electrical, mechanical, and thermal properties [16,17,18]. Single-walled CNTs (SWCNTs) and multi-walled carbon nanotubes (MWCNTs) are the two main types of carbon nanotubes [19]. Additionally, carbon nanotubes can be modified by covalent or non-covalent functionalization, resulting in the introduction of carboxyl (–COOH), hydroxyl (–OH), and carbonyl (–C=O–) groups. Surface-functionalized carbon nanotubes can improve dispersion performance and provide reaction sites to broaden their application range [20]. Magnetic solid-phase extraction (MSPE), a new mode of SPE, is a pretreatment method based on a magnetic adsorbent. The magnetic adsorbent used in MSPE does not need to be packed in any type of device and can be collected easily by an extra magnetic field. Due to the introduction of a magnetic adsorbent, the MSPE procedure became simpler. In addition, high recovery, simple separation, less solvent consumption, and easy surface functionalization of adsorbent are other characteristics of MSPE [21]. In recent years, the applications of MSPE in the analysis of drugs from different matrices have been reported [22,23,24,25,26].

In this work, magnetic tetraethylenepentamine (TEPA)-modified carboxyl–carbon nanotubes (Mag-CCNT-TEPA) were synthesized by a one-pot reaction based on a previously published solvothermal method [27]. In this method, the carboxyl–carbon nanotubes can provide a large number of reaction sites, and magnetic nanoparticles can provide a simple and quick magnetic separation at the same time. MSPE based on using Mag-CCNT-TEPA as adsorbent followed by HPLC with UV detection was developed to extract, separate, and determine seven local anesthetic drugs (procaine, lidocaine, mepivacaine, oxybuprocaine, bupivacaine, tetracaine, and cinchocaine) from human plasma.

## 2. Results and Discussion

### 2.1. Preparation and characterization of Mag-CCNT-TEPA

**Figure 1:**
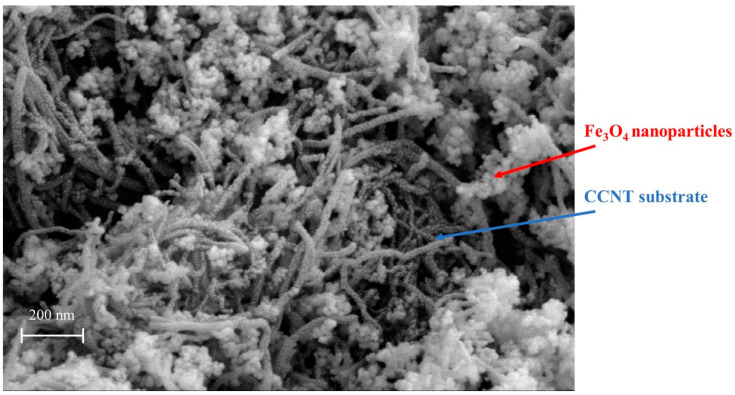
The SEM image of Mag-CCNT-TEPA.

**Figure 2:**
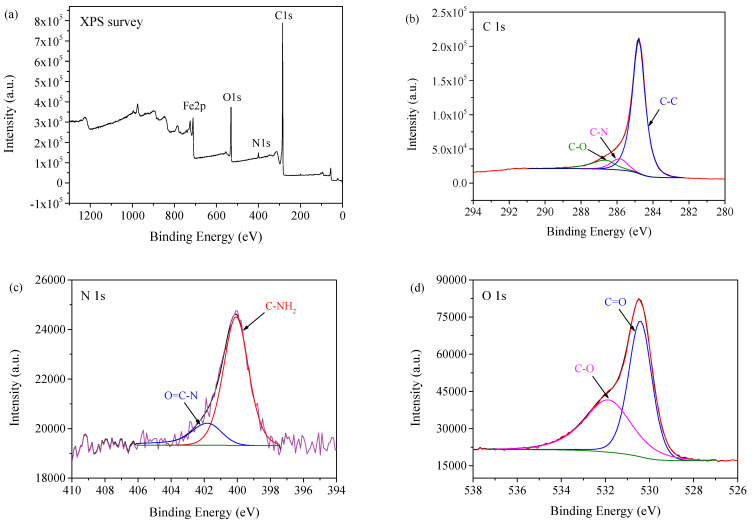

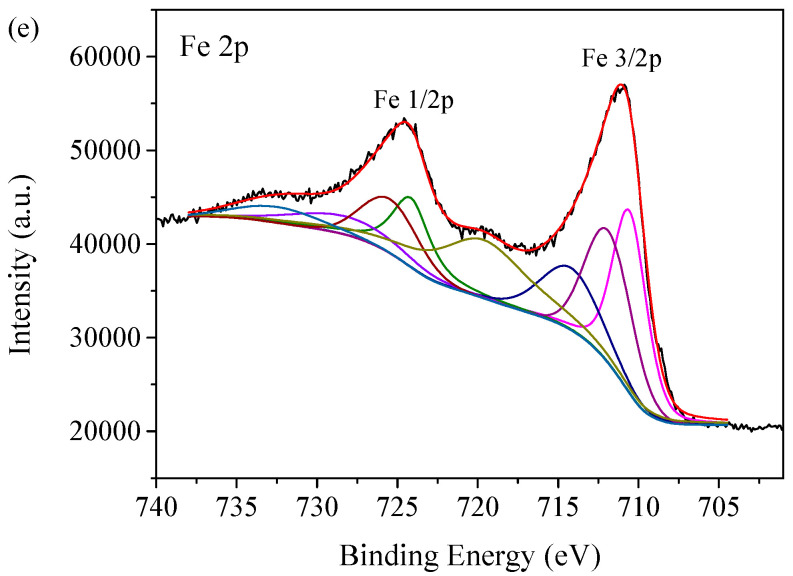
X-ray photoelectron spectroscopy (XPS) analysis of Mag-CCNT-TEPA: XPS survey (**a**), C 1s (**b**), N 1s (**c**), O 1s (**d**), and Fe 2p (**e**).

The morphological characterization of Mag-CCNT-TEPA was conducted by SEM, as shown in Figure 1. It could be observed in the SEM image that the Fe_3_O_4_ nanoparticles had immobilized on the CCNT substrates. XPS was utilized to determine the chemical composition of Mag-CCNT-TEPA, and the results are shown in Figure 2. As we can see in Figure 2a, the characteristic signals detected at 285.0 eV, 530.8 eV, and 711.2 eV were attributed to carbon (C 1s), oxygen (O 1s), and Ferrum (Fe 2p), respectively. At 400.2 eV, a peak was observed, which was attributed to nitrogen (N 1s) introduced by the -NH and -NH_2_ groups from TEPA. The spectra of C1s, N1s, O1s, and Fe2p are shown in Figure 2. Four peaks observed in C1s spectra (Figure 2b) corresponded to C–C (284.80 eV), C–N (285.84 eV), and C–O (286.62 eV). Two peaks observed in N1s spectra (Figure 2c) corresponded to C–NH_2_ (400.07 eV) and O=C–N (401.80 eV). As shown in the O1s spectra (Figure 2d) and Fe2p spectra (Figure 2e), two peaks at the position of 530.41 eV and 531.86 eV belonged to C=O and C–O, and two peaks at the position of 710.99 eV and 724.62 eV belonged to Fe 3/2p and Fe 1/2p. The results were proof of successful TEPA modification, amino functionalization, and magnetic nanoparticle immobilization.

**Figure 3:**
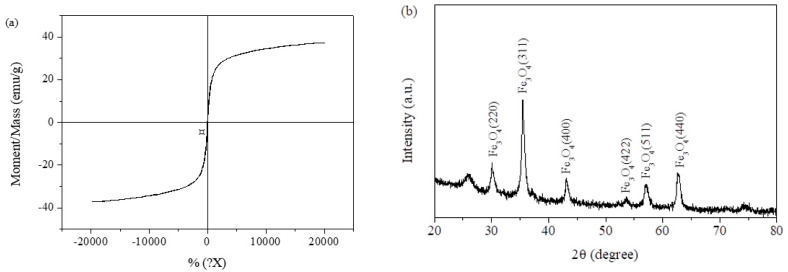
VSM curve (**a**) and XRD pattern (**b**) of Mag-CCNT-TEPA.

The magnetic hysteresis loop analysis conducted by VSM is shown in Figure 3a. The result illustrated that the saturation value of Mag-CCNT-TEPA was 37.2 emu/g. The introduction of CCNT and TEPA did not have a significant impact on the paramagnetic properties of Fe_3_O_4_ nanoparticles, so this material can also provide rapid magnetic separation. The XRD patterns of Mag-CCNT-TEPA (Figure 3b) showed that six characteristic diffraction peaks of Fe_3_O_4_ were 30.1°, 35.5°, 43.1°, 53.6°, 57.1°, and 62.6° indicating that chemical modification did not change the crystal phase property of Fe_3_O_4_.

In conclusion, the successful preparation of Mag-CCNT-TEPA was proven by the characterization conducted by SEM, XPS, VSM, and XRD.

### 2.2. Optimization of Extraction Conditions

In order to obtain maximum extraction recovery, several influential factors, including the usage amount of adsorbent, extraction time, sample pH, and elution conditions, were investigated. Samples spiked at a concentration of 1.0 mg/L were used to optimize extraction conditions.

#### 2.2.1. The Effect of the pH of Sample Solutions

**Figure 4:**
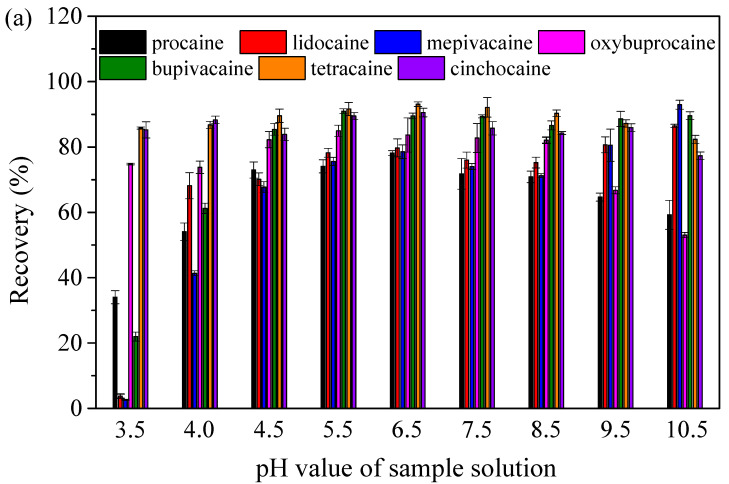

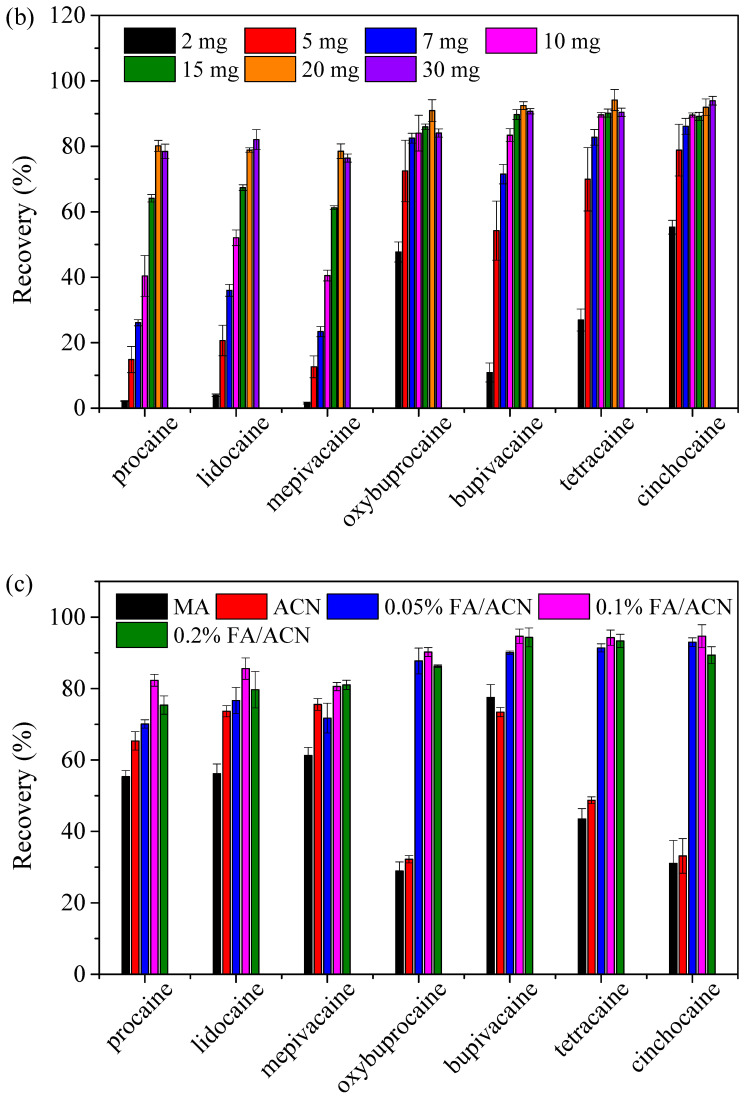
Effects of pH value of sample solution (**a**), adsorbent amount (**b**), and elution solvent (**c**) on the recoveries of seven analytes.

An important factor with a remarkable impact on the adsorption of analytes was the pH value of sample solutions. Various kinds of interactions existed between the analytes and adsorbents, including π–π, hydrogen bond, dipole–dipole, and hydrophobic interaction. The effect of pH on extraction efficiency was considered for the different interactions between the analytes and adsorbents at different pH values. Therefore, the pH value of the sample solution ranging from 3.5 to 10.5 was optimized. The pH of sample solutions was adjusted by sodium hydroxide and hydrochloric acid. As shown in Figure 4a, the results showed that the recoveries of seven local anesthetic drugs were improved with pH values of sample solutions in the range of 3.5–6.5. High recoveries of most of the studied local anesthetic drugs were obtained with a pH of 6.5, ranging from 78.2 to 93.1%. According to the results of the experiments, the pH value of sample solutions was set as 6.5 for further studies. The pH of spiked human plasma was 6.5, so HCl and NaOH were not used for pH adjustment. Different interactions between analytes and adsorbents at different pH values lead to a difference in extraction efficiency. At a low pH, the protonation of amino groups happened, so the amino groups of adsorbents and local anesthetic drugs were transformed into -NH_3_^+^ groups, and π–π interaction played a major role in the extraction. With the increase in the pH value, deprotonation happened gradually, and hydrogen bond and dipole–dipole interaction contributed to the extraction of local anesthetic drugs. Additionally, the remaining unreacted carboxyl groups on the surface of Mag-CCNT-TEPA had electrostatic interaction with the local anesthetic drugs under suitable conditions. However, when the pH value increased excessively, the hydrogen bond and dipole–dipole interaction between the analytes and adsorbents could be disturbed, resulting in a decrease in recoveries. Therefore, in order to obtain satisfactory recoveries of studied analytes, the pH value was fixed at 6.5 for further experiments.

#### 2.2.2. Effect of the Amount of Adsorbents

The amount of adsorbents was a vital factor that affected the extraction recoveries in the MSPE procedure. In order to investigate the effect of the amount of adsorbent, seven different masses ranging from 2 to 30 mg were chosen to further experiments (Figure 4b). The results showed that the recoveries of seven local anesthetic drugs increased with the increase in the amount of adsorbent (2–20 mg). This can be explained that with the increase in the amount of adsorbent, the active sites on the adsorbents increased. When the adsorbent amount achieved 20 mg, satisfactory recoveries of analytes were obtained in the range of 78.9–96.0%. When further increasing the amount of adsorbent, no significant improvements in the recoveries of the seven local anesthetic drugs were observed. The amount of adsorbent was fixed at 20 mg for experiments.

#### 2.2.3. Effect of the Extraction Time

The effect of extraction time on the extraction recoveries was studied in the range of 1–30 min. As results shown in Figure S2a from Supplementary Materials, as the extraction time increased from 1 min to 30 min, the recoveries of analytes increased. Satisfactory recoveries of seven local anesthetic drugs were obtained at 20 min. With the extention of the extraction time, the recoveries do not increase much, but it extends the pretreatment time. In order to obtain satisfactory recoveries and reduce the pretreatment time, the extraction time was fixed at 20 min for further experiments.

#### 2.2.4. Effects of the Elution Conditions

In the MSPE procedure, elution conditions such as the kind and volume of elution solvent and elution time played a crucial role in the elution efficiency. Methanol (MA) and acetonitrile (ACN) were chosen as elution solvents at first. Based on the results (Figure 4c), acetonitrile has a better elution capacity than methanol, so acetonitrile was chosen to further experiments. Because the amino groups could be easily protonated in acidic acetonitrile, formic acid was added to acetonitrile to improve the elution efficiency of the local anesthetic drugs studied. The effect of the formic acid ratio ranged from 0.05% to 0.2% (*V*/*V*) in acetonitrile as the elution efficiency was optimized. The results of the effect of elution solvent are shown in Figure 4c. By increasing the ratio of formic acid in acetonitrile, the concentration of hydrogen ions in acetonitrile increased, and the protonation of amino groups of analytes and adsorbents occurred, resulting in the damage of hydrogen bonds and an increase of elution efficiency. When the ratio of formic acid was 0.1%, good recoveries (80.6–94.6%) of studied local anesthetic drugs were obtained. There was no significant change in recoveries with a further increase in the formic acid ratio. Finally, 0.1% formic acid/acetonitrile (*V*/*V*) was selected as the elution solvent.

Furthermore, 0.25–1.5 mL of 0.1% formic acid/acetonitrile (*V*/*V*) was utilized to evaluate the effect of the elution solvent volume, and the effect of elution time was studied in the range of 1–20 min. The results shown in Figures S2b and S2c from Supplementary Materials indicate that 1.0 mL 0.1% formic acid/acetonitrile (*V*/*V*) and 10 min of the elution time were appropriate to obtain a higher elution efficiency. Therefore, 1.0 mL and 10 min were selected as the elution solvent volume and elution time, respectively.

### 2.3. Regeneration and Reusability of Adsorbent

In order to evaluate the regeneration and reusability of adsorbent, after each absorption, the Mag-CCNT-TEPA-absorbed was regenerated with 5 mL of 1% ammonia solution/acetonitrile and 20 mL (10 mL × 2) of deionized water. The recoveries of analytes higher than 70% obtained by recycled adsorbent were considered acceptable. As shown in Figure S3 from Supplementary Materials, under the condition optimized before, the adsorbent can be reused five times at least with no significant decrease in extraction recovery (72.3–95.4%). Five batches prepared at different times were chosen to evaluate the reproducibility of preparation. The results showed that the synthesis procedure of this material had good reproducibility with relative deviations between batches lower than 4.2%.

### 2.4. Adsorbent Type

The extraction efficiency of Mag-CCNT and Mag-CCNT-TEPA were compared. The results are shown in Figure S4 from Supplementary Materials. Higher recoveries (79.9–95.1%) of seven local anesthetic drugs were obtained by using Mag-CCNT-TEPA as adsorbents. When using Mag-CCNT as the adsorbent, the recoveries of studied analytes decreased to 46.9–82.6%. Well-dispersed carboxyl on the surface of carboxyl–carbon nanotubes provided large amounts of reaction sites with TEPA. The large surface area of multi-walled carboxyl–carbon nanotubes, the increased hydrogen bond between the amino groups (–NH–, –NH_2_) of Mag-CCNT-TEPA introduced by TEPA and local anesthetic drugs, and π–π interaction between the benzene ring of local anesthetic drugs were important factors that enhance the extraction efficiency of local anesthetic drugs.

### 2.5. Method Validation

The method’s validation was determined by the linearity, limit of detection (LOD), limit of quantification (LOQ), and precision. A series of matrix-calibration standards in human plasma with concentrations between 0.02 and 5.00 mg/L were prepared to plot calibration curves. The calibration curves were made by peak areas of analytes against concentration (mg/L). The LOD and LOQ were calculated according to the signal-to-noise (S/N) ratio of 3 and 10. In order to test the precision and accuracy of the proposed method, spiked recoveries at three concentration levels (0.1, 1.0, 4.0 mg/L), intra-day, and inter-day relative standard deviations (RSDs) were conducted by five parallel experiments (*n* = 5). All the results obtained are listed in Table 1. The matrix-calibration curves were linear over a wide concentration range of 0.02–5.00 mg/L, with correlation coefficients (r^2^) values in the range of 0.9980–0.9999. Spiked samples with a low concentration were prepared to determine the LODs and LOQs based on the signal-noise of 3 and 10. The LODs and LOQs values of the seven local anesthetic drugs were achieved in the ranges of 0.003–0.008 mg/L and 0.011–0.028 mg/L. Subsequently, in order to evaluate the accuracy and precision of the proposed method, spiked recoveries and relative standard deviations (RSDs) were determined at three concentration levels (0.1, 1.0, 4.0 mg/L). The intra-day and inter-day RSDs were less than 7.7% and 8.3%, respectively. The mean recoveries of the seven local anesthetic drugs spiked in human plasma varied from 82.0 to 108%. The results of recoveries and RSDs indicated the acceptable precision and high accuracy of this method for the analysis of the seven local anesthetic drugs in human plasma.

In order to assess the matrix effect in human plasma, the matrix-matched calibration curve was established. The matrix effect was calculated based on comparing the slope obtained by the matrix-matched calibration curve and the slope obtained by the standard calibration curve. The results showed that the matrix effects of human plasma on the seven local anesthetic drugs varied in the range of −8.0 to 12.6%. In order to reduce the error caused by the matrix effect, the matrix calibration curve was used for quantitative analysis of seven local anesthetic drugs.

Furthermore, the performance of this proposed method was compared with other reported works [10,11,12,13,14,28,29,30]. The results, including LODs, recoveries, and linearity, are listed in Table 2. The comparison showed that the pretreatment methods for the determination of local anesthetic drugs in biological samples were mostly traditional SPE and LLE [10,11,12,13,28,29,30]. The method established in this chapter applied the magnetic solid-phase extraction method for the determination of anesthetics in plasma. The proposed method had the advantages of high recovery, good linearity, and accuracy. At the same time, the sensitivity of this method is comparable to other reported HPLC-UV [29,30] and GC-MS [28] methods. The LODs of this method cannot be compared to some reported works using mass spectrometry detection. This is mainly because the sensitivity of UV detection is lower than MS. This also provided us with an effective way to improve sensitivity.

### 2.6. Analysis of Real Samples 

**Figure 5:**
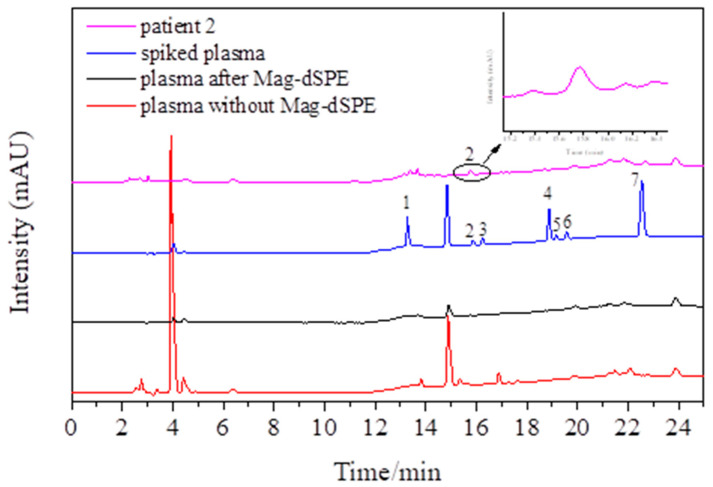
The chromatograms of plasma without MSPE and plasma after MSPE (determined concentration: 0.87 mg/L), spiked plasma (spiked concentration: 1.0 mg/L), and standard in patient no. 2. Peak 1: Procaine, Peak 2: Lidocaine, Peak 3: Mepivacaine, Peak 4: Oxybuprocaine, Peak 5: Bupivacaine, Peak 6: Tetracaine, Peak 7: Cinchocaine.

To further validate this proposed method, the proposed method was further applied to determine the plasma concentration of lidocaine from two patients. Patient no. 1 was treated with 105 mg of lidocaine, 120 mg of propofol, 30 μg of sufentanil, and 50 mg of rocuronium bromide. Patient no. 2 was treated with 100 mg of propofol, 20 μg of sufentanil, 40 μg of remifentanil, 30 mg of rocuronium bromide, and 100 mg of lidocaine for topical throat anesthesia, as well as 300 mg of lidocaine for intratracheal topical anesthesia. The plasma samples were collected at 5, 10, and 30 min after treatment. The lidocaine concentrations in plasma were determined by the proposed method. The lidocaine concentrations in the plasma of patient no. 1 were 1.44, 0.78, and 0.62 mg/L; those in patient no. 2 were 0.39, 0.87, and 1.18 mg/L. The chromatogram of plasma from patient no. 2, the blank plasma without MSPE, the blank plasma with MSPE, and the spiked blank human plasma (spiked concentration: 1.0 mg/L) are displayed in Figure 5. Comparing human plasma before and after the MSPE procedure, a lower signal-to-noise ratio and smaller peaks of interfering substances were observed in the plasma after the MSPE procedure. This result verifies that Mag-CCNT-TEPA can provide a sample cleanup of human plasma to a certain degree. However, interfering substances can also be detected after the MSPE procedure, which means that the selectivity of the Mag-CCNT-TEPA is not very good, and it can also extract other medicine in human plasma during the MSPE procedure. Therefore, in the future, we can modify the adsorbent with specific functional groups to improve selectivity.

## 3. Materials and Methods

### 3.1. Materials and Chemicals

Analytical-grade tetraethylenepentamine (TEPA), sodium acetate anhydrous, and multi-walled carboxyl–carbon nanotubes (o.d. 10–20 nm, length 10–30 μm) were purchased from Aladdin Industrial Corporation (Shanghai, China). Analytical-grade ethylene glycol and ammonium hydroxide (25%) and hydrochloric acid (36%) were purchased from Sinopharm Chemical Reagent Co., Ltd. (Shanghai, China). Analytical-grade ferric chloride hexahydrate (FeCl_3_·6H_2_O), HPLC-grade formic acid (FA), ammonium acetate (AmAc), mepivacaine, bupivacaine hydrochloride, oxybuprocaine, and cinchocaine were purchased from Macklin Biochemical Co., Ltd. (Shanghai, China). Procaine hydrochloride, lidocaine hydrochloride monohydrate, and tetracaine hydrochloride were purchased from Dr. Ehrenstorfer GmbH (Augsburg, Germany). HPLC-grade methanol was purchased from Tedia Company, Inc. (Fairfield, OH, USA).

### 3.2. Equipment

The morphological characterization of Mag-CCNT-TEPA was carried out by scanning electron microscope (SEM, Zeiss Sigma 300, Germany), and the chemical composition was carried out by X-ray photoelectron spectrometer (XPS, Thermo Scientific K-Alpha, USA). The magnetic characteristics of the material were determined by a vibrating sample magnetometer (VSM, LakeShore 7404, USA). The crystal structure characterization was conducted by X-ray diffraction (XRD, Panalytical X’Pert’3 Powder, Germany).

The separation and analysis of seven local anesthetic drugs were performed on an Ultimate 3000 HPLC system equipped with an LPG-3400SD pump, six-port valve with a 20 μL sample loop, TCC-100 thermostat column compartment, and VWD-3400 variable-wavelength UV detector (Thermo Scientific, USA). The deionized water used in all experiments was purified by a water-purification apparatus (Thermo Scientific, USA).

### 3.3. HPLC Analysis

The separation of local anesthetic drugs was performed on a Welchrom C18 (4.6 × 250 mm, 5 μm) column. A total of 5.0 mmol/L AmAc-0.05% formic acid (*v*/*v*) in methanol as eluent A and 5.0 mmol/L-0.05% formic acid (*v*/*v*) in water as eluent B were used as a mobile phase with the following gradient elution: 0–8 min, 12% A; 8–9 min, 12%→20% A; 9–10 min, 20%→40% A; 10–18 min, 40%→80% A; 18–22 min, 80%A; 22–23 min, 80%→12% A. The column oven temperature was fixed at 30 °C. The flow rate and injection volume were 1.0 mL/min and 20.0 μL, respectively. The detection wavelengths were 210 nm (lidocaine, mepivacaine, bupivacaine, and cinchocaine) and 300 nm (procaine, oxybuprocaine, and tetracaine).

### 3.4. Preparation of Mag-CCNT-TEPA

Mag-CCNT-TEPA was prepared by a one-pot reaction. A total of 1.0 g ferric chloride hexahydrate and 3.0 g sodium acetate anhydrous were dissolved in 20 mL ethylene glycol. After that, 5.0 mL ammonium hydroxide was added to the solution. When the solution changed to dark orange, 10.0 mL tetraethylenepentamine was added. Finally, 50.0 mg carboxyl-CNTs was added. All processes underwent ultrasonication. Then, the mixture was allowed to react at 200 °C for 6 h in a 100 mL Teflon-lined stainless-steel autoclave. After the reaction was finished, the black Mag-CCNT-TEPA was washed with deionized water and ethanol several times, dried at 50 °C, and stored in a sealed glass bottle for further use. The schematic diagram of Mag-CCNT-TEPA preparation based on a one-pot reaction is shown in Figure S5 from Supplementary Materials.

### 3.5. Standard Preparation

Local anesthetic drugs were precisely weighted and dissolved in a small amount of methanol and diluted with deionized water to prepare the standard stock solution at the concentration of 1000 mg/L. Additionally, 100 mg/L of the standard solution was prepared by dilution of the stock solutions with deionized water. All stock solutions were sealed and stored in brown glass bottles at 4 °C before analysis.

### 3.6. Application of Mag-CCNT-TEPA for the Extraction of Local Anesthetic Drugs from Human Plasma

Human plasma was provided by Hwa Mei Hospital (Ningbo, China). Patients signed written informed consent before taking part in this study. A total of 1.0 mL plasma was transferred to a 5.0 mL centrifuge tube, and then 2.0 mL methanol was added. The mixtures were extracted under ultrasonication for 5 min, then centrifuged at 15,000 r/min for 6 min. Then, the residue was extracted with 2.0 mL methanol one more time. The supernatants were collected into a 10.0 mL tube for further MSPE procedures.

**Figure 6:**
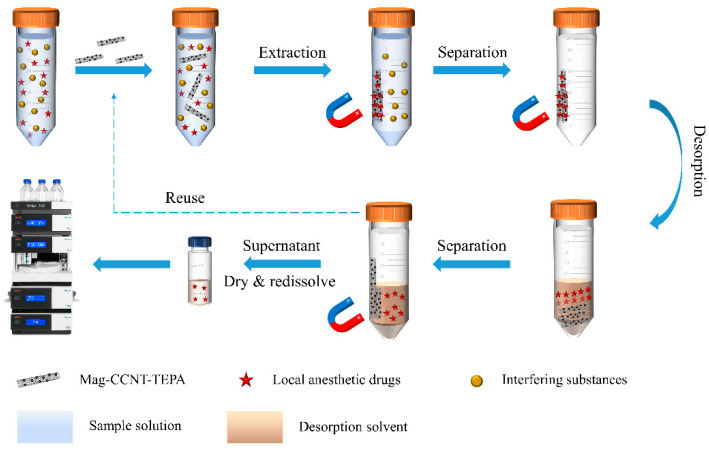
Schematic diagram of the MSPE procedure.

In order to extract local anesthetic drugs in human plasma, 20 mg (accurate to 0.1 mg) Mag-CCNT-TEPA was added to the samples and machine-shaken for 20 min. The adsorbent was then collected by an extra magnetic field and eluted with 1.0 mL 0.1% formic acid/acetonitrile (*V*/*V*). The eluent solution was dried at 40 °C in N_2_ steam and redissolved with 200 μL of an initial mobile phase. The schematic diagram of the MSPE procedure is shown in Figure 6.

## 4. Conclusions

In this work, a simple method for the separation and determination of seven local anesthetic drugs in human plasma was developed based on HPLC coupled with the MSPE procedure. Mag-CCNT-TEPA was successfully synthesized based on a one-pot reaction and exploited as the adsorbent in the MSPE procedure. Magnetic nanoparticles provide a rapid magnetic separation, so they can simplify the sample-preparation procedure. The conditions in the procedures of adsorption and elution were optimized. Under optimal conditions, satisfactory extraction efficiencies of seven local anesthetic drugs were obtained. Parameters including linearity, LODs, recoveries, and RSDs were chosen to evaluate the performance of this proposed method. The results obtained demonstrated that the Mag-CCNT-TEPA-based MSPE method is rapid, sensitive, and suitable for the analysis of local anesthetic drugs in real samples. In the future, improving the selectivity of magnetic adsorbents and combing magnetic solid-phase extraction with MS is needed. 

## Figures and Tables

**Table 1:** Validation parameters, recoveries, and precision of 7 local anesthetic drugs obtained at three concentration levels in blank human plasma.

Compounds	Linear Range (mg/L)	Linear Equation	Correlation Coefficient (r^2^)	Spiked Level (mg/L)	Average Recovery (%)	RSD (%)		Batch–Batch ReProducibility RSD%	LOD (mg/L)	LOQ (mg/L)
						Intra-day	Inter-day			
procaine	0.02–5.00	*Y* = 2.5147*X* − 0.0657	0.9993	0.1 1.0 4.0	82.0 103 100	2.0 2.3 1.6	3.0 7.2 4.6	3.9	0.004	0.014
lidocaine	0.02–5.00	*Y* = 2.7307*X −* 0.0057	0.9995	0.1 1.0 4.0	91.6 93.3 98.9	2.0 5.7 2.8	4.0 3.8 5.4	4.2	0.007	0.025
mepivacaine	0.02–5.00	*Y* = 2.5830*X* + 0.2243	0.9980	0.1 1.0 4.0	97.7 87.7 101	7.1 5.6 4.0	1.7 7.5 4.7	4.0	0.007	0.023
oxybuprocaine	0.04–5.00	*Y* = 2.1061*X* − 0.0648	0.9998	0.1 1.0 4.0	98.7 99.6 94.7	7.7 1.5 1.5	6.0 2.5 2.3	1.5	0.008	0.028
bupivacaine	0.02–5.00	*Y* = 2.7547*X +* 0.0792	0.9998	0.1 1.0 4.0	89.4 93.7 99.3	4.7 6.3 5.1	8.3 4.4 2.3	3.5	0.006	0.022
tetracaine	0.02–5.00	*Y* = 3.9807*X +* 0.0555	0.9998	0.1 1.0 4.0	82.8 90.0 94.7	1.7 1.9 1.7	1.5 1.6 1.8	1.9	0.004	0.015
cinchocaine	0.02–5.00	*Y* = 7.3979*X* − 0.0917	0.9999	0.1 1.0 4.0	91.1 108 108	1.8 2.5 4.3	2.6 2.3 2.6	3.3	0.003	0.011

**Table 2:** Comparison of the analytical features of MSPE-based methodologies for the determination of local anesthetic drugs.

Analysis	Preparation Method	Samples	Analytes	Correlation Coefficient (r^2^)	LOQ (µg/L)	Recovery (%)	Ref.
LC-MS/MS	SPE	Human serum	Nine local anesthetic drugs	>0.9858	10	81.4–144	[10]
HPLC-MS/MS	LLE	Human plasma	Lidocaine	>0.9996	1.0	95.2–104	[11]
LC-MS/MS	LLE	Human plasma	Bupivacaine	>0.998	0.25	/	[12]
LC-MS/MS	LLE	Blood	Two local anesthetic drugs	>0.9991	0.1–0.8	85.4–87.5	[13]
LC-MS/MS	MEPS	Human plasma	Two local anesthetic drugs	>0.995	0.44–0.47	91.0–118	[14]
GC-MS	SPE	Plasma and urine	Seven local anesthetic drugs	>0.983	50–100	73.0–95.0	[28]
HPLC-UV	LLE	Human serum	Lidocaine	0.9995	43	80.4–93.9	[29]
HPLC-UV	LLE	Human plasma	Five local anesthetic drugs	>0.9980	50	91.7–106	[30]
HPLC-UV	MSPE	Human plasma	Seven local anesthetic drugs	>0.9980	11–28	82.0–108	This work

## Data Availability

Details are available from authors.

## Supplementary Materials

The following supporting information can be downloaded at: https://www.mdpi.com/article/10.3390/molecules27175509/s1, Figure S1. Chemical structures of seven local anesthetic drugs; Figure S2. The effect of extraction time (a), desorption solvent volume (b), desorption time (c); Figure S3. The effect of extraction cycles of extraction efficiency; Figure S4. Comparison of extraction efficiency correlated with recovery of 7 local anesthetic drugs using Mag-CCNT and Mag-CCNT-TEPA; Figure S5. The schematic diagram of Mag-CCNT-TEPA preparation.

## Sample Availability

Samples of the compounds are available from the authors.
