# Supplementary material for: Determination of Local Anesthetic Drugs in Human Plasma Using Magnetic Solid-Phase Extraction Coupled with High-Performance Liquid Chromatography
Source: Molecules. 2022 Aug 27;27(17):5509. doi: 10.3390/molecules27175509 (PMC9457896; doi:10.3390/molecules27175509)
Supplement: Supplementary file 1 [file molecules-27-05509-s001.zip › molecules-1868341-supplementary.pdf]

Supplementary Materials

Determination of Local Anesthetic Drugs in Human Plasma Using Magnetic Solid-Phase Extraction Coupled with High-Performance Liquid Chromatography

Shan-Yan Liang [1,†], Fang Shi [2,†], Yong-Gang Zhao [3,*,†] and Hong-Wei Wang [4,*,†]

[1] Hwa Mei Hospital, University of Chinese Academy of Sciences, Ningbo 315010, China
[2] Department of Chemistry, Zhejiang University, Hangzhou 310027, China
[3] College of Biological and Environmental Engineering, Zhejiang Shuren University, Hangzhou 310015, China
[4] Tongde Hospital of Zhejiang Province, Hangzhou 310012, China
* Correspondence: zhyg91213@163.com (Y.-G.Z.); wanghw1022@163.com (H.-W.W.)
† These authors contributed equally to the work.

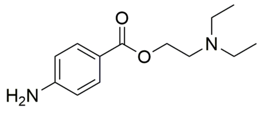
procaine

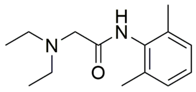
lidocaine

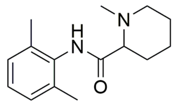
mepivacaine

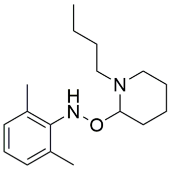
bupivacaine

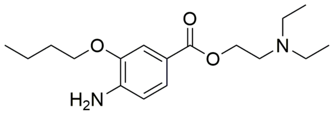
oxybuprocaine

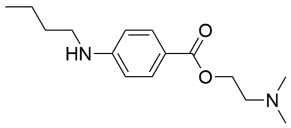
tetracaine

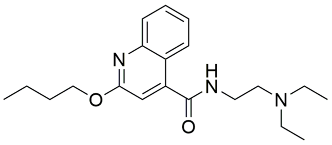
cinchocaine

Figure S1. Chemical structures of seven local anesthetic drugs.

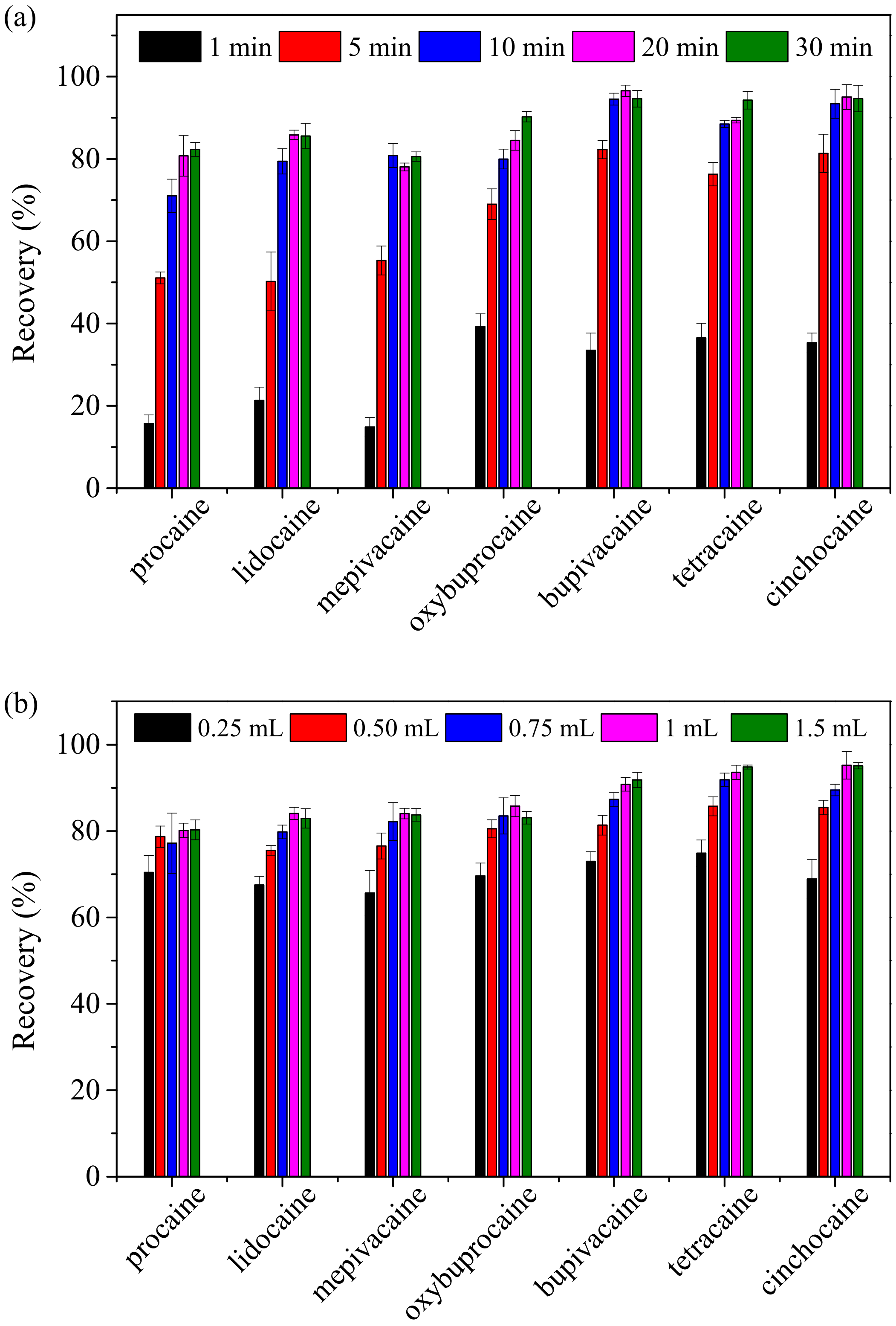
(a)
1 min
5 min
10 min
20 min
30 min
Recovery (%)
0
20
40
60
80
100
procaine
lidocaine
mepivacaine
oxybuprocaine
bupivacaine
tetracaine
cinchocaine
(b)
0.25 mL
0.50 mL
0.75 mL
1 mL
1.5 mL
Recovery (%)
0
20
40
60
80
100
procaine
lidocaine
mepivacaine
oxybuprocaine
bupivacaine
tetracaine
cinchocaine

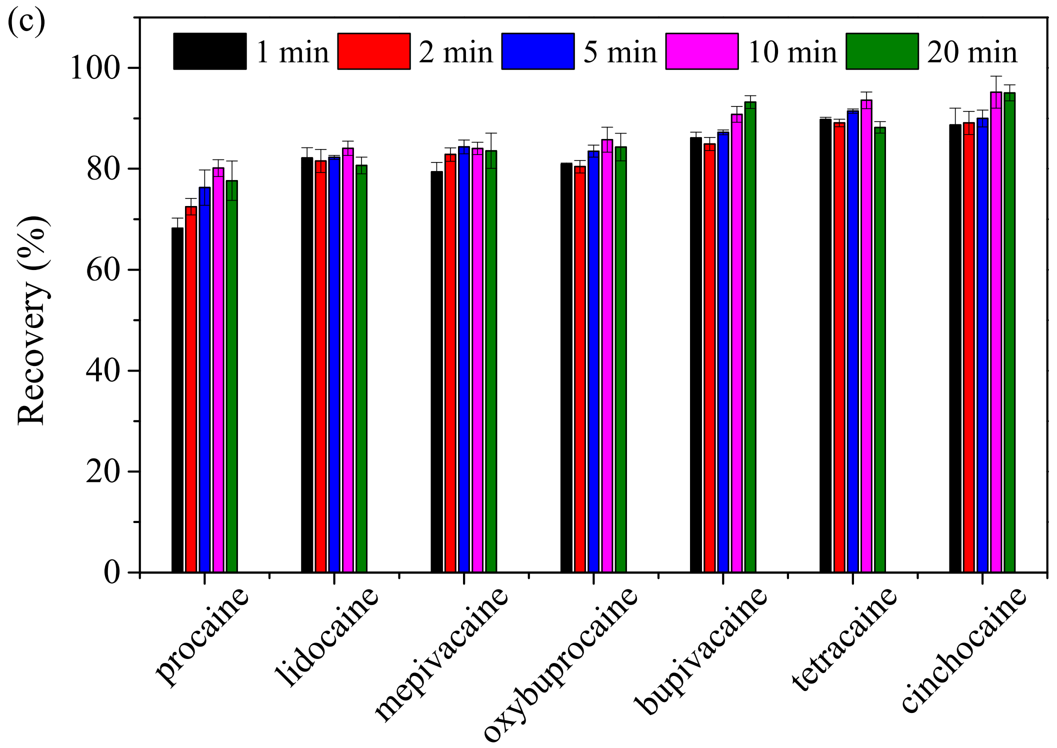

Figure S2. The effect of extraction time (a), desorption solvent volume (b), desorption time (c),

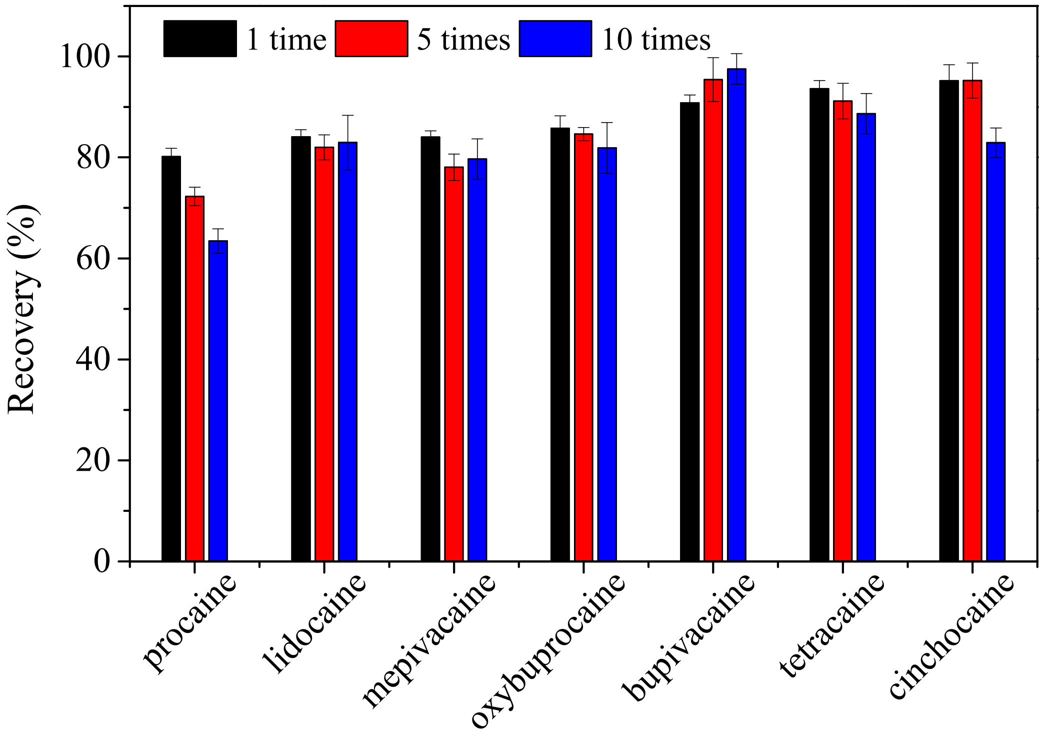

Figure S3. The effect of extraction cycles of extraction efficiency.

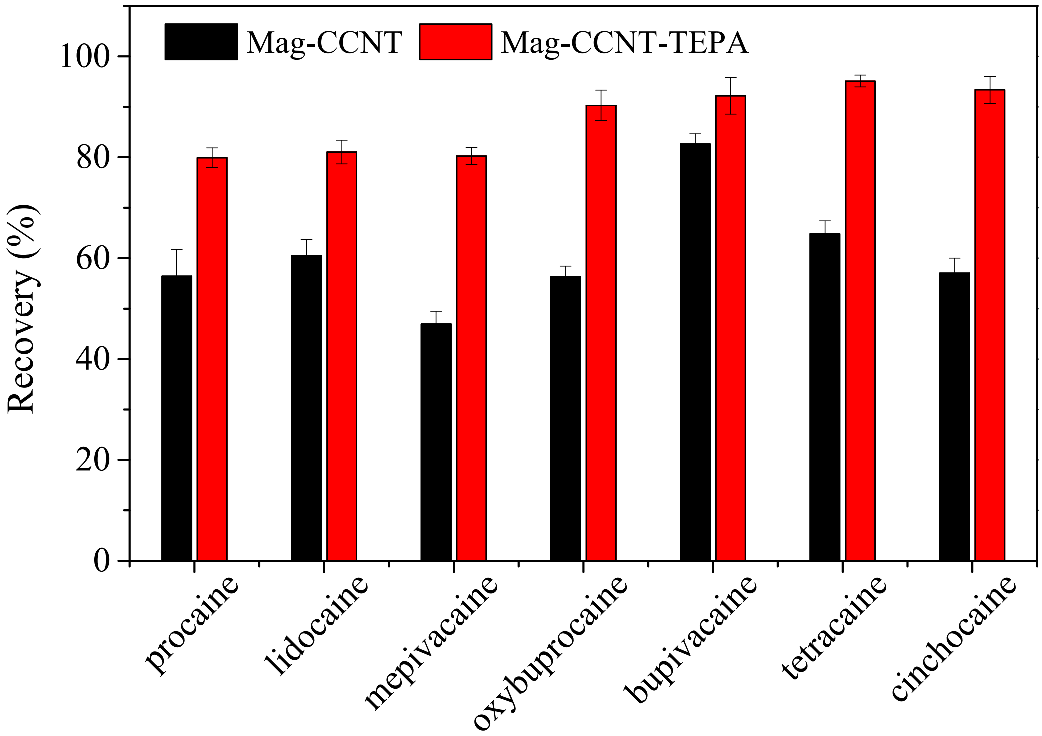

Figure S4. Comparison of extraction efficiency correlated with recovery of 7 local anesthetic drugs using Mag-CCNT and Mag-CCNT-TEPA.

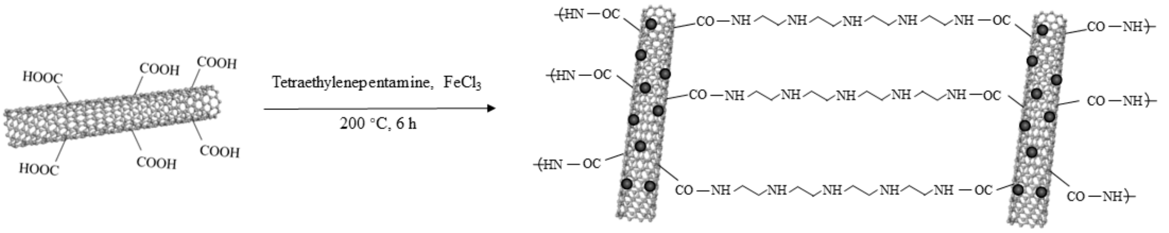

Figure S5. The schematic diagram of Mag-CCNT-TEPA preparation.
